# First report of *Serratia marcescens* associated with black rot of *Citrus sinensis *fruit *,* and evaluation of its biological control measures in Bangladesh

**DOI:** 10.12688/f1000research.27657.2

**Published:** 2022-03-04

**Authors:** Mohammed Faruk Hasan, Mohammed Asadul Islam, Biswanath Sikdar

**Affiliations:** 1 Genetic Engineering and Biotechnology, University of Rajshahi, Rajshahi, 6205, Bangladesh; 2 Professor Joarder DNA and Chromosome Research Lab., Department of Genetic Engineering and Biotechnology, University of Rajshahi, Rajshahi, 6205, Bangladesh

**Keywords:** Orange, Black rot, Serratia marcescens, 16S rRNA gene, Biological control

## Abstract

**Background:** The present study was designed to isolate and identify the phyto-pathogen responsible for black rot of *Citrus sinensis*, and to determine its biological control measures.

**Methods:** The pathogen was isolated from infected oranges and cultured on Luria-Bertani medium. Gram staining method was used to identify the morphological characteristics of the causal agents of the black rot. Advanced molecular technique was applied to facilitate proper detection of the isolated bacteria. Phylogenetic trees were analyzed using the Neighbor-Joining method. Antimicrobial screening was conducted by disc diffusion method. Antagonistic activity was evaluated by well diffusion method.

**Results:** Gram staining of the causal agent showed rod shaped, small and pink bacteria. Polymerase chain reaction of the 16S ribosomal RNA gene amplified an approximately 1465 bp product. The nucleotide sequences of the isolated bacterial sample 1 (BS1) and bacterial sample 2 (BS2) had 99.34% and 99.45% similarities with the reference of *Serratia marcescens* sequence in NCBI GenBank. The obtained sequences were deposited in GenBank. Two isolates showed virulence capability on some fresh fruits, which confirmed the stain detection and Koch’s postulates. *Allium sativum* extract showed the largest (27.33±1.5 mm) diameter of zone of inhibition against BS1, at 30µg/disc concentration. In the antagonistic assay, *Rhizobium leguminosarum* showed largest (19±1 mm) zone of inhibition against BS1.

**Conclusions: **Findings of the current investigations are constructive for identification of causative pathogens in *Citrus sinensis* black rot disease and their biological control measures.

## Introduction

Orange, *Citrus sinensis* (L.) Osbeck is the most common and important species among citrus, belonging to the family of Rutaceae. Orange peel has significant contents of vitamin C, dietary fiber, total polyphenols, carotenoids, limonene and dietary minerals ( Barros *et al*., 2012). Conventionally, oranges fruits are used to treat disorders like constipation, pains, indigestion, diarrhea, bronchitis, tuberculosis, cough, cold, obesity, menstrual disorders, angina, hypertension, nervousness, downheartedness and pressure ( Milind & Chaturvede, 2012).

*Serratia marcescens* is an ecologically and genetically diverse pathogenic bacteria species belonging to the family of Enterobacteriaceae ( Besler & Little, 2017). *S. marcescens* may be present as a contamination on the surface of plants or fruits and enter the tissue via natural pores or sores ( Wang *et al*., 2015). Previous reports showed that this bacteria is an opportunist pathogen that causes diseases in humans, such as cystitis ( Liu *et al*., 2004), conjunctivitis ( Hume & Willox, 2004) and others.

*Serratia marcescens* has been reported as a plant pathogen causing yellow vine disease in *Citrullus lanatus* and *Cucurbita pepo* ( Sikora *et al*., 2012), soft rot in *Capsicum annuum* ( Gillis *et al*., 2014), *Zea mays* whorl rot ( Wang *et al*., 2015), yellow wilt on *Helianthus* ( Ignatov *et al*., 2016), leaf rot in *Cannabis sativa* ( Schappe *et al*., 2020) and others. Naturally active plant products are important source of eco-friendly bactericides to control different pathogenic bacteria ( Rahman *et al*., 2014).

To the best of our knowledge, there is no previous report in Bangladesh on black rot disease of *Citrus sinensis* fruit, including isolating the pathogen and identifying it through molecular approaches. Moreover, biological control of this pathogen by plants extract and antagonistic microbes has not yet been reported.

Therefore, the objective of this study was to isolate and characterize phytopathogenic bacterial strains responsible for black rot of orange, as well as their controlling techniques using biological agents. The 16S rRNA gene sequence analysis and pathogenicity assays were performed to determine the taxonomic position of the isolated bacterial strains. 

## Methods

### Isolation and purification of bacteria

In 2018 (September-December), eight black rotted orange fruits were collected from RDA market, Rajshahi, Bangladesh. Bacterial pure colonies were isolated and purified from infected fruits as described previously ( Lu & Gross, 2010). Briefly, the infected skin of the fruits were excised with a sterile scalpel and disinfected superficially using 70% alcohol for one minute, sodium hypochlorite for one minute, and finally was rinsed three times in sterile distilled water. The samples were then placed in 100 ml of Luria-Bertani broth medium and incubated at 37°C overnight. Bacteria was streaked onto a fresh LB agar plate and incubated for 18 hours at 37°C. After that, eight different single colonies were picked up by loop and streaked on fresh medium plate to obtained pure culture. Finally, two pure cultures were selected and preserved in 80% glycerol-water solution at -80°C. The two pure cultures were characterized and identified using different approaches, as below.

### Morphological characterization

Gram staining and motility of bacteria was conducted as previously described ( Zaoti *et al*., 2018).

### Molecular characterization

The two bacterial isolates were sub-cultured on LB liquid medium and incubated at 30°C for 16 hours with continuous shaking. Genomic DNA was extracted using an automated DNA extractor (Model: Maxwell 16; Promega, USA) and suspended into TE buffer. Afterwards, quantity and quality of the isolated DNA was checked using NanoDrop Spectrophotometer (Model: ND2000; Thermo Scientific, USA).

The isolated bacterial DNA was amplified using the universal bacterial 16S rRNA gene PCR primers (27F 5’-AGAGTTTGATCCTGGCTCAG3’ and 1492R 5’-CGGTTACCTTGTTACGACTT-3’), as previously described ( Srinivasan *et al*., 2015; Tian *et al*., 2019); genomic DNA was used as a template. The PCR reaction was performed using the method by Hassan *et al*. (2018) and Hasan *et al*. (2020), using hot start green master mix (dNTPs, Buffer, MgCl _2_, Taq Pol; Cat: M7432; Promega, USA).

A 20 µl reaction mix was used, containing master mixture 10 µl, T DNA (concentration 25–65 ng/µl) 1 µl, each primer (concentration 10–20 pMol) 1 µl and nuclease free water 7 µl. Thermo-cycling parameters were 95°C for 3 minutes, 32 cycles of 95°C for 30 seconds, 48°C for 30 seconds and 72°C for 90 seconds; a final extension step at 72°C was added for 5 minutes, using a PCR machine (Gene Atlas, Model: G2; Astec, Japan). PCR products were run on 1% agarose gel (Cat: V3125; Promega, USA) and visualized under alpha imager UV trans-illumination (Model: mini; Protein Simple, USA) with 0.5% ethidium bromide solution (Cat: H5041; Promega, USA) in 1xTAE buffer (Cat: V4251; Promega, USA) using a1kb DNA ladder (Cat: G5711; Promega, USA). PCR products were purified from the agarose gel, using SV gel and PCR clean up system (Cat: A9281; Promega, USA). Purified PCR products were sequenced commercially by Sanger sequencing (Apical Scientific, Malaysia). Sequences were used in a search using the NCBI BLAST tool. Obtained sequences were submitted to GenBank (see *Data* availability) and compared with the GenBank database.

### Evolutionary relationships of taxa

A phylogenetic tree was constructed using the Neighbor-Joining method ( Saitou & Nei, 1987) based on the 16S rRNA gene. The bootstrap consensus tree inferred from 500 replicates is taken to represent the evolutionary history of the taxa analyzed. Branches corresponding to partitions reproduced in less than 50% bootstrap replicates are collapsed. The percentage of replicate trees in which the associated taxa clustered together in the bootstrap test (500 replicates) are shown next to the branches ( Felsenstein, 1985). The evolutionary distances were computed using the Maximum Composite Likelihood method ( Tamura *et al*., 2004) and are in the units of the number of base substitutions per site. All ambiguous positions were removed for each sequence pair (pairwise deletion option). Evolutionary analyses were conducted in MEGA X ( Kumar *et al*., 2018).

### Pathogenicity assay

Virulence competencies of the isolates were carried out as previously described by Tafinta *et al.* (2013). First, healthy, fresh, mature malta, lemon, guava and apple fruits were collected from the RDA market, Rajshahi and were sterilized using water and 70% ethanol. The two isolates were grown on LB agar, suspended in autoclaved distilled water at a concentration of 10 ^8^ cells/ml ( Ignatov *et al*., 2016) and artificially inoculated into malta, lemon, guava and apple fruits. Autoclaved distilled water was treated as a negative control. All the inoculated fruits showed an oversensitive response after 16 hours. To fulfill Koch’s postulates, bacteria were re-isolated from the artificially infected fruits tissue showing symptoms, and characteristics were confirmed based on colony morphology ( Abdellatif *et al*., 2015) on LB agar along with PCR results.

### *In* vitro antimicrobial screening of the plant extracts

To evaluate antibacterial activities of the isolates, *Allium sativum, Hibiscus rosa-sinensis, Ocimum sanctum, Allium cepa, Zingiber montanum* and *Psidium guajava* plants were collected from various places (Kajle and Binodpur villages) in Rajshahi district. Plants extraction was performed using the method described by Kader *et al*. (2018). Different parts of selected plants, bulb of *A. sativum* and *A. cepa*; flowers of *H. rosa-sinensis;* and leaves of *O. sanctum, Z. montanum,* and *P. guajava* were cut into small pieces, air-dried, and ground by blender to form a fine powder. The dried powder of the selected plants (100gm of each plant) were rinsed in methanol (500ml) using a conical flask and kept in a shaking incubator for 15 days. The liquid contents were pressed through Markin cloth followed by filtration using Whatman no. 1 filter paper. Obtained filtered liquids were dehydrated in vacuo to leave a blackish and sticky mass. The extracts were preserved in a refrigerator at 4°C using glass vials.

For antibacterial screening, BS1 and BS2 bacterial cultures were inoculated on LB agar plates. Subsequently, discs of filter paper (6 mm in diameter) were saturated with the plant extracts at the concentration of 30 µg/disc and were impregnated on the surface of plates. The plates were incubated at 37°C for 18 hours. Inhibition zones were measured with the help of a millimeter scale.

### Antagonistic control

For evaluation of antagonistic effects, five soil bacteria, *Rhizobium phaseoli, Rhizobium leguminosarum, Escherichia coli, Bacillus subtilis* and *Brevibacillus borstelensis* were used against isolated bacterial strains. Pure cultures of the soil bacteria were kindly provided by Dr. Md. Salah Uddin, Associate Professor and Director, Microbiology Lab., Department of Genetic Engineering and Biotechnology, University of Rajshahi, Rajshahi, 6205, as the part of collaboration.

To assess the antagonistic effects, agar well diffusion method was used, as previously described ( Valgas *et al*., 2007). The agar plate’s surface was inoculated by spreading a volume of the microbial inoculum over the entire agar surface. A 6 mm diameter hole was made aseptically with a cork borer. In total, 40 µl/hole bacterial suspension (10 ^8^ CFU/ml) of the isolates were introduced into the well and were incubated at 37°C for 18 hours. The antagonistic soil bacteria were diffused in the LB agar medium that inhibit the growth of two isolated plant pathogens. The test samples were determined by measuring the diameter of zones of inhibition in term of mm with a transparent scale.

### Data analysis

All the experiments and test were done triplicate. All data were expressed as the mean value and standard deviation (M ± SD) using Microsoft Excel software 2013.

## Results

### Morphological characterization of isolates

**Figure 1.**
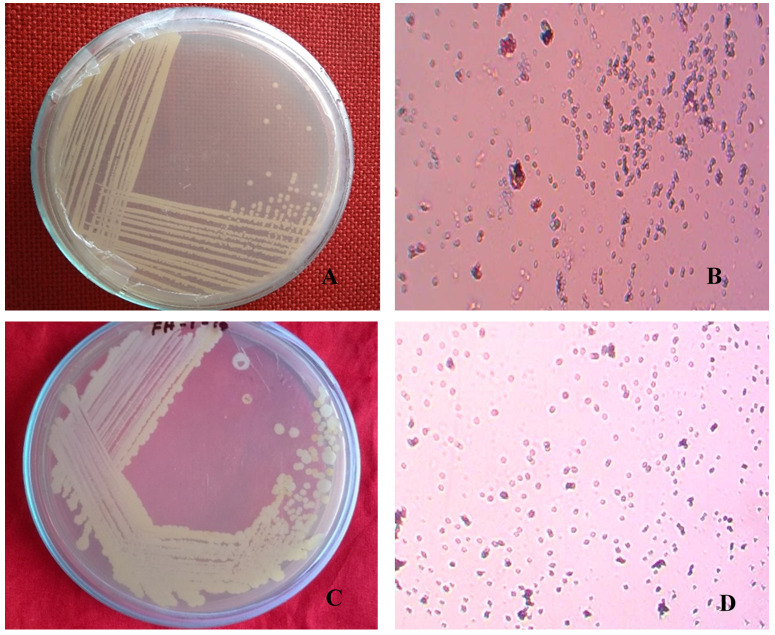
Naturally infected postharvest sweet orange fruit showing pure culture and morphological phenotypes. Isolated bacterial pure colony ( **A**) BS1 and ( **C**) BS2; images taken after 18 hours of incubation at 37°C on LB agar. Gram staining of isolated bacterial pure colony ( **B**) BS1 and ( **D**) BS2; images taken under the light microscope at 100X magnification, showing as Gram negative.

Single and pure colonies were isolated from the infected fruit grown in LB agar medium. The isolated colonies (BS1 and BS2) were round and pure cultures showed cream and white colonies ( Figure 1A and C, respectively). Gram stained BS1 and BS2 showed small, rod shaped, pink, motile and Gram-negative colonies ( Figure 1B and D).

### Molecular characterization of isolated bacteria

Electrophoretic analysis of the DNA isolated from the bacterial strain revealed sharp high molecular weight of the DNA band, which indicated that the DNA was of good quality and suitable for PCR analysis. From the electrophoretic analysis approximately 1465 bp of PCR product was observed while the negative control did not show any product ( *Underlying data* ( Hasan, 2020b)).

### Evolutionary relationships of taxa

**Figure 2.**
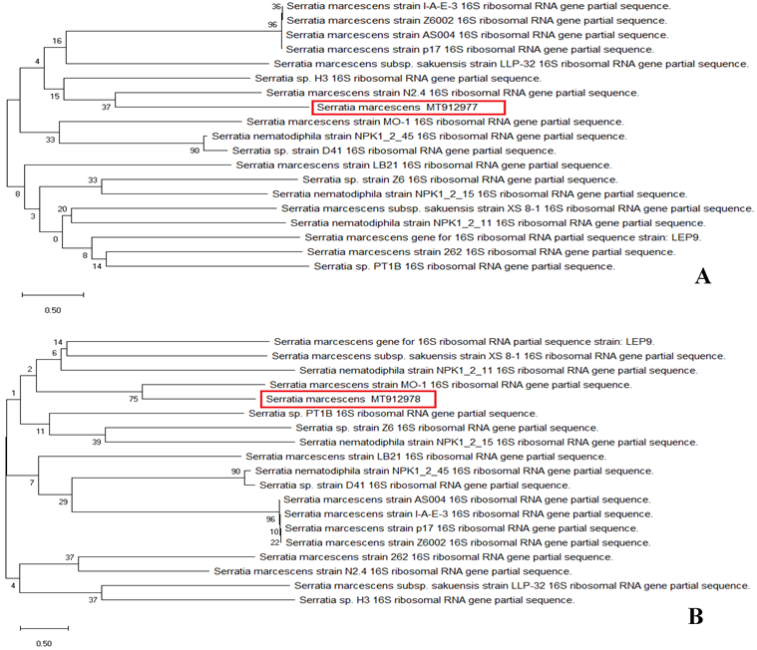
Evolutionary relationships of taxa. Phylogenetic tree of ( **A**) BS1 and ( **B**) BS2. The evolutionary history was inferred using the Neighbor-Joining method ( Saitou & Nei, 1987). This analysis involved 21 nucleotide sequences. Codon positions included were 1st+2nd+3rd+Noncoding. There were a total of 1494 positions in the final dataset. The scale bar on the rooted tree indicates a 0.50 substitution per nucleotide position.

The sequences of the total isolates (BS1 and BS2) were compared to *Serratia marcescens* sequences in GenBank using BLASTN. The partial 16S rRNA gene sequences (accession no. MT912977 and MT912978) were 99.34% and 99.45% ( Figure 2A and B), identical to the reference sequence for *S. marcescens* strains (ID: AY514434.1) and (ID: JX315621.2), respectively.

### Pathogenicity assay

**Figure 3.**
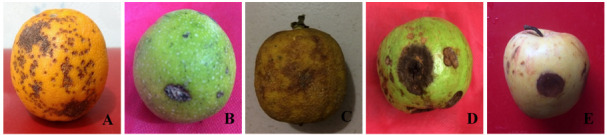
Naturally infected and artificially inoculated fruit showing symptoms of bacterial black rot. ( **A**) post-harvest natural infection, ( **B**) malta (BS1), ( **C**) lemon (BS1), ( **D**) guava (BS2), and ( **E**) apple (BS2) (artificially inoculated four fruits); images taken 10 days after inoculation.

The pathogenicity assay showed similar morphological characteristics of *Serratia marcescens* causing black rot symptoms ( Figure 3A–E). Isolated bacterial DNA from artificially inoculated malta, lemon, guava and apple fruits showed approximately 1465 bp PCR products ( *Underlying data* ( Hasan, 2020d)).

### *In vitro* antimicrobial screening of the plant extracts

**Figure 4.**
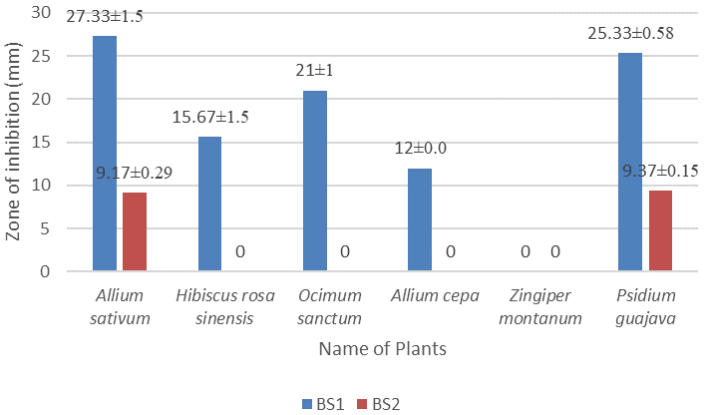
Antibacterial activities of *Allium sativum, Hibiscus rosa sinensis, Ocimum sanctum, Allium cepa, Zingiper montanum,* and *Psidium guajava* plants extract against the isolates BS1 and BS2. Methanol was used as solvent, bacteria was cultured in LB, plated were incubated at 37°C for 18 hours.

The methanol extract of *Allium sativum* showed the largest diameter of zone of inhibition (27.33±1.5 mm and 9.17±0.29 mm) against the isolates BS1 and BS2, respectively, at 30 µg/disc concentrations. *Zingiber montanum* leaf extract didn’t show any antibacterial activity against the isolates BS1 and BS2. Data for all plants are given in Figure 4 and in *underlying data* ( Hasan, 2020e; Hasan, 2020f; Hasan, 2020g).

### Antagonistic activity of soil bacteria against the isolates

**Figure 5.**
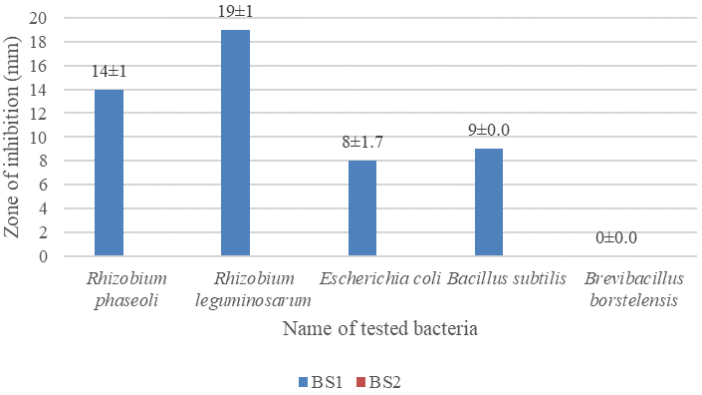
Antagonistic activity of soil bacteria, *Rhizobium phaseoli, Rhizobium leguminosarum, Escherichia coli, Bacillus subtilis* and *Brevibacillus borstelensis* against the isolates BS1 and BS2 (it was done in Figure 4). Isolates were grown on LB agar; test bacteria were cultured and incubated at 37°C for 18 hours.

The largest inhibition zone was found to be 19±1 mm for *Rhizobium leguminosarum* followed by 14±1 mm for *Rhizobium phaseoli* against the isolated bacteria. *Brevibacillus borstelensis* showed no zone of inhibition against the isolated bacteria (images are given in *underlying data* ( Hasan, 2020i; Hasan, 2020j)). Data are given in Figure 5.

## Discussion

Morpho-molecular identification has been used as universal adequate procedure to detect bacterium through all key phyta. Evaluation of the bacterial 16S rRNA gene, sequencing has developed as a favored genetic method ( Clarridge, 2004). Now a day, it has also developed as a significant technique to identify an unfamiliar bacterium to the genus or species level ( Sacchi *et al*., 2002). To distinguish this new disease from the identified rot diseases stated earlier, we propose ‘black rot’ as the common name for the orange disease.

In the present study, the isolated bacterial strains were revealed as gram negative and motile. Tian *et al*. (2019) reported that *Serratia marcescens* is a rod-shaped, gram negative bacterium isolated from waste samples which support our present findings. PCR and sequencing was accomplished by 16S rRNA gene amplification for molecular detection of the isolated bacterial strains. Wang *et al.* (2015) found an identity of 99% to *Serratia marcescens* strain CPO1 by the BLAST search. Similar result was also reported previously by Altschul *et al.* (1990). Pathogenicity efficiency of the isolates were confirmed the stains detection using malta, lemon, guava and apple fruits. No significant resistance to this bacterial pathogen was observed in the investigation of the tested fruits. Moreover, *Allium sativum* plant extract and *Rhizobium leguminosarum* showed remarkable inhibitory potency against BS1. Villar de Queiroz & Soares de Melo (2006) reported that the strain R3.5 of *S. marcescens* has an antagonist to *P. parasitica in vitro*. Present findings revealed the isolates as *Serratia marcescens* strain, which is responsible for black rot disease of *Citrus sinensis* fruits.

## Conclusions

Bacterial black rot reduces the quantity and quality of the orange and cause chronic losses to the farmers and fruit industries. Advanced molecular approaches along with morphological characterization confirmed the identity of the bacteria as *Serratia marcescens.* In order to biological control measures of the isolated bacteria, *Allium sativum* plant extract and *Rhizobium leguminosarum* bacteria showed promising results. In conclusion, *Serratia marcescens* causing black rot of orange fruit is a new disease in Bangladesh. Further investigation is needed to better comprehend the biology of the isolates, disease development procedure, and to develop orange varieties resistant or biological control to the bacterial disease in near future.

## Data availability

### Underlying data

*Serratia marcescens* strain BS1 containing 16S rRNA gene and partial sequence on GenBank. Accession number, MT912977: https://www.ncbi.nlm.nih.gov/nuccore/MT912977.1/

*Serratia marcescens* strain BS2 containing 16S rRNA gene and partial sequence on GenBank. Accession number, MT912978: https://www.ncbi.nlm.nih.gov/nuccore/MT912978.1/

Figshare: Naturally infected postharvest sweet orange fruit showing pure culture and morphological phenotypes, https://doi.org/10.6084/m9.figshare.13108142 ( Hasan, 2020a).

This project contains the following underlying data:

- Original, unedited images as shown in Figure 1

Figshare: PCR amplification of 16S rRNA gene generated from bacteria, https://doi.org/10.6084/m9.figshare.13140074 ( Hasan, 2020b).

This project contains the following underlying data:

- Original, unedited gel image for BS1 (left lane) and BS2 (right lanes) isolates

Figshare: Naturally infected and artificially inoculated sample fruits showing symptoms of bacterial black rot, https://doi.org/10.6084/m9.figshare.13109120.v3 ( Hasan, 2020c).

This project contains the following underlying data:

- Original, unedited images as shown in Figure 3

Figshare: PCR amplification of 16S rRNA gene generated from bacteria for virulence test, https://doi.org/10.6084/m9.figshare.13140017 ( Hasan, 2020d).

This project contains the following underlying data:

- Original, unedited gel images for BS1 isolate, and infected malta, lemon, guava and apple fruits (left to right lanes)

Figshare: Antibacterial activities of methanolic extract of some plant against the isolates BS1 and BS2, https://doi.org/10.6084/m9.figshare.13160348 ( Hasan, 2020e).

Figshare: Antibacterial activities of some plants extract against the isolate BS1, https://doi.org/10.6084/m9.figshare.13160615 ( Hasan, 2020f).

This project contains the following underlying data:

- Images of zone of inhibition of BS1 for *Allium sativum, Hibiscus rosa-sinensis, Ocimum sanctum, Allium cepa, Zingiber montanum* and *Psidium guajava*

Figshare: Antibacterial activities of some plants extract against the isolate BS2, https://doi.org/10.6084/m9.figshare.13160648 ( Hasan, 2020g).

This project contains the following underlying data:

- Images of zone of inhibition of BS2 for *Allium sativum, Hibiscus rosa-sinensis, Ocimum sanctum, Allium cepa, Zingiber montanum* and *Psidium guajava*

Figshare: Antagonistic activity of soil born bacteria against the isolates BS1 and BS2, https://doi.org/10.6084/m9.figshare.13139981.v1 ( Hasan, 2020h).

Figshare: Antagonistic activity of soil born bacteria against the isolates BS1, https://doi.org/10.6084/m9.figshare.13108055 ( Hasan, 2020i).

This project contains the following underlying data:

- Images of zone of inhibition of BS1 for *Rhizobium phaseoli, Rhizobium leguminosarum, Escherichia coli, Bacillus subtilis* and *Brevibacillus borstelensis.*

Figshare: Antagonistic activity of soil born bacteria against the isolate BS2, https://doi.org/10.6084/m9.figshare.13108061 ( Hasan, 2020j).

This project contains the following underlying data:

- Images of zone of inhibition of BS2 for *Rhizobium phaseoli, Rhizobium leguminosarum, Escherichia coli, Bacillus subtilis* and *Brevibacillus borstelensis.*

Data are available under the terms of the Creative Commons Attribution 4.0 International license (CC-BY 4.0).

## References

[ref-1] Abdellatif E Kaluzna M Helali F : First report of citrus bacterial blast and citrus black pit caused by *Pseudomonas syringe* pv. *syringae* in Tunisia. *New Disease Rep.* 2015;32(1):35. 10.5197/j.2044-0588.2015.032.035

[ref-2] Altschul SF Gish W Miller W : Basic local alignment search tool. *J Mol Biol.* 1990;215(3):403–410. 10.1016/S0022-2836(05)80360-2 2231712

[ref-3] Barros HR Ferreira TA Genovese MI : Antioxidant capacity and mineral content of pulp and peel from commercial cultivars of citrus from Brazil. *Food Chem.* 2012;134(4):1892–1898. 10.1016/j.foodchem.2012.03.090 23442635

[ref-4] Besler KR Little EL : Diversity of *Serratia marcescens* Strains Associated with Cucurbit Yellow Vine Disease in Georgia. *Plant Dis.* 2017;101(1):129–136. 10.1094/PDIS-05-16-0618-RE 30682311

[ref-5] Clarridge JE 3rd : Impact of 16S rRNA Gene Sequence Analysis for Identification of Bacteria on Clinical Microbiology and Infectious Diseases. *Clin Microbiol Rev.* 2004;17(4):840–862, table of contents. 10.1128/CMR.17.4.840-862.2004 15489351 PMC523561

[ref-7] Felsenstein J : Confidence limits on phylogenies: An approach using the bootstrap. *Evolution.* 1985;39(4):783–791. 10.1111/j.1558-5646.1985.tb00420.x 28561359

[ref-8] Gillis A Rodriguez M Santana M : *Serratia marcescens* associated with bell pepper ( *Capsicum annuum* L.) soft-rot disease under greenhouse conditions. *Eur J Plant Pathol.* 2014;138(1):1–8. 10.1007/s10658-013-0300-x

[ref-9] Hasan M : Naturally infected postharvest sweet orange fruit showing pure culture and morphological phenotypes. *Figshare.* 2020a. 10.6084/m9.figshare.13108142

[ref-10] Hasan M : PCR amplification of 16S rRNA gene generated from bacteria. *Figshare.* 2020b. 10.6084/m9.figshare.13140074

[ref-11] Hasan M : Naturally infected and artificially inoculated sample fruits showing symptoms of bacterial black rot. *Figshare.* 2020c. 10.6084/m9.figshare.13109120.v3

[ref-12] Hasan M : PCR amplification of 16S rRNA gene generated from bacteria for virulence test. *Figshare.* 2020d. 10.6084/m9.figshare.13140017

[ref-13] Hasan M : Antibacterial activities of methanolic extract of some plant against the isolates BS1 and BS2. Dataset. *Figshare.* 2020e. 10.6084/m9.figshare.13160348

[ref-14] Hasan M : Antibacterial activities of some plants extract against the isolate BS1. *Figshare.* 2020f. 10.6084/m9.figshare.13160615

[ref-15] Hasan M : Antibacterial activities of some plants extract against the isolate BS2. *Figshare.* 2020g. 10.6084/m9.figshare.13160648

[ref-16] Hasan M : Antagonistic activity of soil born bacteria against the isolates BS1 and BS2. Dataset. *Figshare.* 2020h. 10.6084/m9.figshare.13139981.v1

[ref-17] Hasan M : Antagonistic activity of soil born bacteria against the isolates BS1. *Figshare.* 2020i. 10.6084/m9.figshare.13108055

[ref-18] Hasan M : Antagonistic activity of soil born bacteria against the isolate BS2. *Figshare.* 2020j. 10.6084/m9.figshare.13108061

[ref-19] Hasan MF Islam MA Sikdar B : First report on molecular identification of *Fusarium* species causing fruit rot of mandarin ( *Citrus reticulata*) in Bangladesh [version 1; peer review: 1 approved]. *F1000Res.* 2020;9:1212. 10.12688/f1000research.26464.1 PMC9971657 36865764

[ref-20] Hassan O Jeon JY Chang T : Molecular and morphological characterization of *Colletotrichum* species in the *Colletotrichum gloeosporioides* complex associated with persimmon anthracnose in South Korea. *Plant Dis.* 2018;102(5):1015–24. 10.1094/PDIS-10-17-1564-RE 30673381

[ref-21] Hume EBH Willox MDP : Emergence of *Serratia marcescens* as an ocular surface pathogen. *Arch Soc Esp Oftalmol.* 2004;79(10):475–481. 15523567

[ref-22] Ignatov AN Khodykina MV Polityko VA : First report of *Serratia marcescens* causing yellow wilt disease on sunflower in Russia. *New Disease Reports.* 2016;33:8. 10.5197/j.2044-0588.2016.033.008

[ref-23] Kader SMA Hasan M Ahmed S : Antioxidant, Antibacterial and Cytotoxic activities of Ethanol extract and its different fractions of *Sterculia cordata* leaves. *Discovery Phytomedicine.* 2018;5(3):26–33. 10.15562/phytomedicine.2018.64

[ref-24] Kumar S Stecher G Li M : MEGA X: Molecular Evolutionary Genetics Analysis across computing platforms. *Mol Biol Evol.* 2018;35(6):1547–1549. 10.1093/molbev/msy096 29722887 PMC5967553

[ref-25] Liu JW Hsu YM Huang YF : Independent prognostic factors for fatality in patients with urinary tract infection caused by *Serratia marcescens*. *Infect Control Hosp Epidemiol.* 2004;25(1):80–82. 10.1086/502297 14756225

[ref-26] Lu SE Gross DC : Drippy pod of white lupine: a new bacterial disease caused by a pathovar of *Brenneria quercina*. *Plant Dis.* 2010;94(12):1431–1440. 10.1094/PDIS-05-10-0365 30743385

[ref-27] Milind P Chaturvede D : Orange: Range of benefits. *International Research Journal of Pharmacy.* 2012;3(7):59–63. Reference Source

[ref-28] Rahman A Islam R Al-Reza SM : *In vitro* control of plant pathogenic *Xanthomonas* spp. using *Poncirus trifoliata* Rafin. *EXCLI J.* 2014;13:1104–1110. 26417325 PMC4464478

[ref-29] Sacchi CT Whitney AM Mayer LW : Sequencing of 16S rRNA gene: a rapid tool for identification of *Bacillus anthracis*. *Emerg Infect Dis.* 2002;8(10):1117–1123. 10.3201/eid0810.020391 12396926 PMC2730316

[ref-30] Saitou N Nei M : The neighbor-joining method: A new method for reconstructing phylogenetic trees. *Mol Biol Evol.* 1987;4(4):406–425. 10.1093/oxfordjournals.molbev.a040454 3447015

[ref-31] Schappe T Ritchie DF Thiessen LD : First Report of *Serratia marcescens* Causing a Leaf Rot Disease on Industrial Hemp ( *Cannabis sativa*). *Plant Disease.* 2020;104(4):1248. 10.1094/PDIS-04-19-0782-PDN

[ref-32] Sikora EJ Bruton BD Wayadande AC : First Report of the Cucurbit Yellow Vine Disease Caused by *Serratia marcescens* in Watermelon and Yellow Squash in Alabama. *Plant Dis.* 2012;96(5):761. 10.1094/PDIS-09-11-0739-PDN 30727534

[ref-33] Srinivasan R Karaoz U Volegova M : Use of 16S rRNA Gene for Identification of a Broad Range of Clinically Relevant Bacterial Pathogens. *PLoS One.* 2015;10(2):e0117617. 10.1371/journal.pone.0117617 25658760 PMC4319838

[ref-36] Tafinta IY Shehu K Abdulganiyyu H : Isolation and Identification of Fungi Associated with the Spoilage of Sweet Orange ( *Citrus sinensis*) Fruits In Sokoto State. *Nigerian Journal of Basic and Applied Science.* 2013;21(3):193–6. 10.4314/njbas.v21i3.4

[ref-34] Tamura K Nei M Kumar S : Prospects for inferring very large phylogenies by using the neighbor-joining method. *Proc Natl Acad Sci U S A.* 2004;101(30):11030–11035. 10.1073/pnas.0404206101 15258291 PMC491989

[ref-35] Tian C Zhao J Zhang Z : Identification and molecular characterization of *Serratia marcescens* phages vB_SmaA_2050H1 and vB_SmaM_2050HW. *Arch Virol.* 2019;164(4):1085–1094. 10.1007/s00705-019-04169-1 30788604

[ref-37] Valgas C Souza SMD Smânia EF : Screening methods to determine antibacterial activity of natural products. *Brazilian J microbial.* 2007;38(2):369–380. 10.1590/s1517-83822007000200034

[ref-6] Villar de Queiroz BP Soares de Melo I : Antagonism of *Serratia marcescens* towards *Phytophthora parasitica* and its effects in promoting the growth of citrus. *Braz J Microbiol.* 2006;37:448–450. 10.1590/S1517-83822006000400008

[ref-38] Wang X Bi T Li X : First Report of Corn Whorl Rot Caused by *Serratia marcescens* in China. *J Phytopathol.* 2015;163(11–12):1059–1063. 10.1111/jph.12366

[ref-39] Zaoti Z Hasan SM Hossain M : Biochemical and Molecular Characterization of Bacterial Wilt Disease of Banana and Evaluation of their Antibiotic Sensitivity. *Microbiol Res J Int.* 2018;22(6):1–10. Reference Source

