# Supplementary material for: First report of Serratia marcescens associated with black rot of Citrus sinensis fruit , and evaluation of its biological control measures in Bangladesh
Source: F1000Res. 2022 Mar 4;9:1371. Originally published 2020 Nov 26. [Version 2] doi: 10.12688/f1000research.27657.2 (PMC8581594; doi:10.12688/f1000research.27657.2)
Supplement: Peer review history

# Peer review: Reviewer response for version 2

**DOI:** 10.5256/f1000research.79529.r126312

**Published:** 2022-03-07

**Authors:** Ciamak Ghazaei

**Affiliations:** 1 Department of Microbiology, University of Mohaghegh Ardabili, Ardabil, Iran

All changes and the revision are approved.

 Sincerely,

 Ciamak Ghazaei

Is the work clearly and accurately presented and does it cite the current literature?

Partly

If applicable, is the statistical analysis and its interpretation appropriate?

Not applicable

Are all the source data underlying the results available to ensure full reproducibility?

Yes

Is the study design appropriate and is the work technically sound?

Yes

Are the conclusions drawn adequately supported by the results?

Yes

Are sufficient details of methods and analysis provided to allow replication by others?

Partly

Reviewer Expertise:

Microbiology

I confirm that I have read this submission and believe that I have an appropriate level of expertise to confirm that it is of an acceptable scientific standard.

# Peer review: Reviewer response for version 1

**DOI:** 10.5256/f1000research.30572.r98171

**Published:** 2021-11-10

**Authors:** Ciamak Ghazaei

**Affiliations:** 1 Department of Microbiology, University of Mohaghegh Ardabili, Ardabil, Iran

This manuscript does an appropriate demonstrating significant bacterial strain by isolation and identification a phytopathogen. This research is quite an interesting round up of the "First report of Serratia marcescens associated with black rot of *Citrus sinensis* fruit...".

 Whilst the information presented is generally suitable, the level of some presentation limits the ease of digestion of the content. In general, the manuscript would benefit from the clearing of sentences as this avoids misunderstanding of facts. Also the authors could consider a susceptibility test (Antibiotic resistance) comparison for isolates of *Serratia marcescens* which would be more attractive to the readership.

 All the queries and concerns are remarked within the manuscript file.

Is the work clearly and accurately presented and does it cite the current literature?

Partly

If applicable, is the statistical analysis and its interpretation appropriate?

Not applicable

Are all the source data underlying the results available to ensure full reproducibility?

Yes

Is the study design appropriate and is the work technically sound?

Yes

Are the conclusions drawn adequately supported by the results?

Yes

Are sufficient details of methods and analysis provided to allow replication by others?

Partly

Reviewer Expertise:

Microbiology

I confirm that I have read this submission and believe that I have an appropriate level of expertise to confirm that it is of an acceptable scientific standard, however I have significant reservations, as outlined above.

# Response

**Published:** 2021-11-15

**Authors:** Md. Faruk Hasan

**Affiliations:** University of Rajshahi, Bangladesh

We corrected the manuscript according to the reviewer comments and suggestions. We also deleted and added some words and sentence following reviewer suggestions.

# Peer review: Reviewer response for version 1

**DOI:** 10.5256/f1000research.30572.r81642

**Published:** 2021-04-15

**Authors:** Mapotso Kena

**Affiliations:** 1 Department of Plant Production, Soil Science and Agricultural Engineering, University of Limpopo, Rolokwane, South Africa

The authors mentioned several methods being used for morphological identification, however, only two methods were used and this must be corrected. Reference to "plant extracts" as biological control is wrong and this must be rectified by the authors.

 This further affect the study title which should be modified to accomodate plant extracts. 

 It is necessary to also explain in detail how inoculation was done for both isolates.

 Under " *In vitro* antimicrobial screening of the plant extracts", the second but last sentence of the last paragraph needs re-phrasing.

 Clarity is needed with some figures and results presentation and this has been highlighted in the text. Discussion can be improved with additional reference to existing information on biological control and use of plant extracts in the control of other pos-harvest diseases.

 Some suggested minor editorial corrections are highlighted in the text.

Is the work clearly and accurately presented and does it cite the current literature?

Yes

If applicable, is the statistical analysis and its interpretation appropriate?

Yes

Are all the source data underlying the results available to ensure full reproducibility?

Yes

Is the study design appropriate and is the work technically sound?

Partly

Are the conclusions drawn adequately supported by the results?

Partly

Are sufficient details of methods and analysis provided to allow replication by others?

Yes

Reviewer Expertise:

Plant Pathology, biological control of plant pathogens, identification and characterization.

I confirm that I have read this submission and believe that I have an appropriate level of expertise to confirm that it is of an acceptable scientific standard.

# Response

**Published:** 2021-11-15

**Authors:** Md. Faruk Hasan

**Affiliations:** University of Rajshahi, Bangladesh

We read through the manuscript and corrected it according to the reviewer comments.

# Peer review: Reviewer response for version 1

**DOI:** 10.5256/f1000research.30572.r78843

**Published:** 2021-02-19

**Authors:** Kator Liamngee

**Affiliations:** 1 Department of Biological Sciences, Benue State University, Makurdi, Nigeria

The work is clear, accurately presented, and cites current literature.

 The study design is not properly presented. There should be a subheading under the in vitro antimicrobial screening of the plant extracts bearing experimental design. The factors in the experiment should be clearly brought out, treatment combinations and the number of units/lots should be clearly mentioned so one can understand the experimental layout/arrangement.

 The work is technically sound but details of methods are not sufficient to allow replications. This is shown in the pathogenicity. The author needs to explain in details how the two isolates, BS1 and BS2 grown on LB agar, were artificially inoculated into Malta, lemon, guava, and apple fruits during the pathogenicity assay; and the nature of the data collected.

 The concentrations of the plant extracts used in the in vitro antimicrobial screening should be clearly stated with clear descriptions on how the concentrations were arrived at.

 There should be an experimental design for the antagonistic control experiment. The factors in the experiment should be spelt out, treatment combinations (if any) should be mentioned and number of units or lots clearly stated to boost understanding of the arrangement or layout of this particular experiment.

 Sources of data underlying the results are available. The statistical analysis and its interpretation is appropriate and all conclusions are adequately supported by the results.

Is the work clearly and accurately presented and does it cite the current literature?

Yes

If applicable, is the statistical analysis and its interpretation appropriate?

Yes

Are all the source data underlying the results available to ensure full reproducibility?

Yes

Is the study design appropriate and is the work technically sound?

Partly

Are the conclusions drawn adequately supported by the results?

Yes

Are sufficient details of methods and analysis provided to allow replication by others?

Partly

Reviewer Expertise:

Postharvest Biology, Plant pathology, Postharvest pathology, Biocontrol using plant extracts.

I confirm that I have read this submission and believe that I have an appropriate level of expertise to confirm that it is of an acceptable scientific standard, however I have significant reservations, as outlined above.

# Response

**Published:** 2021-02-26

**Authors:** Md. Faruk Hasan

**Affiliations:** University of Rajshahi, Bangladesh

I am approving the reviewer comments and suggestions.

# Response

**Published:** 2021-11-15

**Authors:** Md. Faruk Hasan

**Affiliations:** University of Rajshahi, Bangladesh

I am approving the reviewer comments and suggestions.
